# What Do Israeli Gerontology Students Think About Medical Marijuana Use for Alzheimer’s and Parkinson’s Disease?

**DOI:** 10.1093/geroni/igab046.1354

**Published:** 2021-12-17

**Authors:** Offer Edelstein, Richard Isralowitz, Oren Wacht, Alexander Reznik, Yaacov Bachner

**Affiliations:** 1 Ben-Gurion University of the Negev, Beer-Sheva, HaDarom, Israel; 2 Ben-Gurion University of the Negev, Be'er-Sheva, HaDarom, Israel; 3 Ben-Gurion University, Beer-Sheva, HaDarom, Israel

## Abstract

Aims: The aims of the current study were as follows: 1) to assess gerontology graduate students’ beliefs about medical marijuana’s (MMJ) effectiveness for two common age-related conditions - Alzheimer’s (AD) and Parkinson’s disease (PD); 2) to assess students’ beliefs and attitudes toward MMJ; 3) to explore associations linking background characteristics, MMJ-related attitudes and beliefs, and beliefs about the MMJ effectiveness for AD and PD. Method: A sample of 104 (84 women and 20 men) gerontology graduate students voluntarily participated in an anonymous online survey. Results: The vast majority (95%) of the participants indicated they had no formal education about MMJ and reported being unprepared to answer clients’ MMJ-related questions (84.6%). Most of the participants believed that MMJ is effective for use with AD (70.2%) and PD (80.8%) patients. Participants reported favorable beliefs about MMJ benefits, concerns about risks, the need for training, and positive attitudes toward recreational marijuana use legalization. Prior marijuana use (e.g., self-use, friends or family) was found to be associated with more positive beliefs about MMJ benefits, risks, and its legalization for recreational purposes. Prior marijuana use was the only factor associated with the belief that MMJ is an effective therapy for use with individuals diagnosed with AD or PD. Conclusions: The study findings stress the need for students’ MMJ education in order to provide future gerontology service providers with the necessary knowledge and ability to address clients’ questions about MMJ use. Efforts to develop curricula and training programs need to be promoted.

